# Notes and News

**Published:** 1899-06-10

**Authors:** 


### Notes and News.

(Collected and Communicated.)

At the dinner of the Royal Institute of Public Health the gold medal was presented to Lord Lister.

Another outbreak of typhus fever has occurred in the same quarter of Edinburgh as before, and is reported to be due to dirt and overcrowding.

Scarlet fever has broken out on the warship "Curacoa" in Campbelltown Harbour. A little camp has been formed on shore, where the crew will live in tents whilst the ship is disinfected.

Mr. Lidbrooke F. Cope, of St. George's Hospital, London, has been awarded the British Medical Temperance Association's prize, open to fourth year students in the United Kingdom, for an essay on "The Dietetic Value of Alcoholic Liquors and their Action on the Digestive Organs."

The first volume of a great work, which is to be known by the title of the "Encyclopædia Medica," is to make its appearance this month. It is being published by Messrs. William Green and Sons, of Edinburgh; the London agents being Messrs. J. and A. Churchill. The intention is that it shall not be restricted to pure medicine, but shall cover the entire field of medicine, surgery, midwifery, and the specialities.

In Rome a school for children of weak intellect has been working for some months. It has been established by the Society for the Medico-Pedagogic treatment of the mentally deficient, and is under the charge of Professor de Sanctis, an aurist, and two consultants. The age of the pupils varies from six to 11, and all are those who have been dismissed from private or public schools on account of their mental or normal defects.

The Government is about to establish and maintain three hospitals in the State of West Virginia, America. These hospitals are for accident cases occurring from injuries incurred on railways and in coal mines, and the institutions are to be placed conveniently near a railway. Such hospitals are called miners' hospitals, and free treatment is given to employés in mines and on railways and to labourers, and after this other persons can be admitted at a cost proportionate to their means.

The Queen has forwarded a donation of £25 to the secretary of the British Home for Incurables, in response to his appeal, in aid of the Completion Fund for the erection of a new wing, the foundation-stone of which was laid by the Earl Amherst on May 30th.

Dr. J. Buckley Bradbury, professor of medicine at Cambridge, will deliver the Croonian Lectures before the Royal College of Physicians, on June 20th, 22nd, 27th, and 29th, at five o'clock, the subject being on "Sleep, Sleeplessness, and Hypnotics."

The deadly effect of sewer gas has been unhappily illustrated at Wigan, where three men descended one after another through a man trap to the town sewer, and were overcome before being able to utter a warning. A man who descended in search of the others narrowly escaped the same fate.

With our special Hospital Sunday Supplement we this week present a plate containing portraits of Royal patrons of the London hospitals. There never was a period when members of our Royal Family devoted more of their time either to pleading the claims of our charitable institutions or to attending functions organised on their behalf; and we are sure that our readers will appreciate this addition to the Supplement.

The annual meeting of the Royal British Medical Association will be held this year at Dover, under the presidency of Professor Michael Facer. The locality is full of scientific interest. The character of the meeting is likely to be more international than usual, as the Belgian biologists will pay a visit to Dover, and the French Association for the Advancement of Science, holding its meeting also in September at Boulogne, will probably be entertained by the association.

The Lord Mayor has issued a powerful appeal on behalf of the Hospital Sunday Fund, and many of the clergy have done the same individually to their congregations. It is, therefore, to be hoped that all will join hands on the present occasion to respond to these efforts being made to augment the resources of the hospitals. Mr. George Herring has led the way by his magnificent donation of £10,000, which he gives in testimony of his admiration of the methods adopted by the Metropolitan Hospital Sunday Fund.

Strong complaints have been made to the vestry of St. Pancras respecting the insanitary condition, mainly due to overcrowding, in that district. The vestry formed a committee to inquire into the matter, and the overcrowding was admitted, and the medical officer of health comes in for the main share of the blame. He is declared to have exercised very little, if any, systematic inspection, and he is to be asked to relinquish a post of lecturer to Guy's Hospital which he holds, that he may give more time to St. Pancras. It seems hardly necessary to employ an officer of so high a standing to collect what are merely statistics respecting so shifting a population as the very poor, unless he has the power to promptly secure that the evicted shall obtain proper accommodation afterwards. Very often the lodger is regarded as the evil to be removed; but, as the lodger pays mostly half the rent, his removal necessitates that of the family also, to still more crowded quarters. Altogether, it is hard that a medical officer should be regarded as mainly responsible, when often he feels he can do little more than report.

